# Use of Disposable Punch Biopsy Device to Add Foley Catheter Fenestration to Improve Drainage of Post Radical Prostatectomy Anastomotic Leak

**DOI:** 10.51894/001c.7024

**Published:** 2019-03-04

**Authors:** Aubrey Allen, Jason Wynberg, Eric Walton

**Affiliations:** 1 Detroit Medical Center Department of Urology, PGY2 Resident; 2 Specialist in Chief, Detroit Medical Center Department of Urology; 3 Wayne State Medical School, 3rd year medical student

**Keywords:** fenestrated catheter, urine leak, anastomotic leak, radical prostatectomy

## Abstract

**CONTEXT:**

Radical prostatectomy (RP) is a major oncologic urological surgery that can have high morbidity if complications arise. Bladder-urethral urine anastomotic leaks (AL) are one of the most common complications and can greatly increase morbidity. To date, there are few resources to manage AL. One management technique is using a Foley catheter with an additional auxiliary drainage port, also known as a fenestrated catheter. This type of auxiliary drainage port allows a low-pressure drainage source that is located near the anastomosis to increase urine drainage from catheter rather than from the AL site. The optimal size and location of this additional drainage port is currently unknown. This experiment evaluated the optimal auxiliary drainage port size and an inexpensive technique to easily construct such a catheter.

**METHODS:**

Utilizing different size punch biopsies, auxiliary drainage ports were placed in different size Foley catheters and drainage rates and the structural integrity of the catheter was assessed.

**RESULTS:**

A 3.0 mm punch biopsy located 1.0 cm proximal to the Foley balloon in an 18 French (Fr) catheter was determined to be the optimal size. A 2.0 mm punch biopsy provided significantly less drainage. The 4.0 mm punch biopsy compromised the structural integrity of the catheter.

**CONCLUSIONS:**

Based on these experimental results, we recommend using a 3.0 mm punch biopsy in an 18 Fr catheter 1.0 cm. proximal to the balloon for an auxiliary drain site in Foley catheter when the anastomosis is not watertight or the surgeon has reason to believe the patient is at higher risk for an AL Factors such as history of pelvic radiation, abnormal anatomy, large prostate, post-surgical hematoma formation, obesity, previous prostatic surgery, difficult anastomosis, blood loss and postoperative urinary tract infection may make use of this type of device more attractive.

## INTRODUCTION

Prostate cancer is the most common cancer in men and third most common cancer overall with 161,360 new diagnosis and 26,730 deaths in 2017.^1^ About 60,000 radical prostatectomies (RP) are annually performed in the US.^2^ The primary indication for RP is almost always adenocarcinoma of the prostate. This oncologic surgery includes removing the prostate and seminal vesicles and suturing the bladder neck to the urethra to form the anastomosis (i.e., the connection made between the bladder and urethra).

An indwelling urethral catheter is then left in place to allow the anastomosis to heal. This anastomosis can occasionally leak urine into the tissues surrounding the bladder and is one of the most feared post-operative complications from a RP. The published rate of anastomotic leak (AL) following radical prostatectomy is 0.3 to 15.4%,^3^ meaning that approximately 180 to 9,240 prostate cancer patients in the US are annually affected by AL.

AL has been associated with multiple complications including extended hospital stays, ileus (i.e., lack of intestinal movement), infection and possibly increased risk of bladder neck contracture and/or permanent incontinence.^4^ In addition, urine is irritating to intra-abdominal organs and the peritoneal lining. If bacteria are present, the risk for infection is a significant concern that can cause sepsis, abscess formation and further tissue and organ damage.^4^ An AL is traditionally managed by an intra-abdominal drain and extended Foley catheter drainage. If a urine leak is recognized post-operatively and no drain had been left, an interventional radiology procedure to place a drain is required.^4^

A less well-known technique includes using an intra-abdominal drain and a fenestrated Foley catheter that consists of a second drainage hole, usually placed on the other side of the inflation balloon from the standard drainage hole.^5^

Adding an auxiliary drainage port to the catheter provides any drainage ports on both sides of the balloon a competing drainage site for AL urine flow as this hole is closer to the anastomosis and ureteral orifice than the standard distal catheter holes.^5,6^ Fenestrated catheters have been used for decades in the management of AL. Multiple case reports and one randomized control trial have demonstrated that fenestrated catheters can significantly reduce anastomotic leakage post-operatively.^5–7^ To our knowledge, there have not previously been studies evaluating the optimal size fenestration and catheter size to manage AL flow. As such the purpose of this experiment was to evaluate the optimal auxiliary drainage port size and offer an inexpensive technique to easily construct such a catheter.

## METHODS

For this experiment, a Bard^®^ 18 French (Fr) 5cc balloon Foley catheter (# 0165L18) was utilized. The catheter balloon was filled with 5 ml of water to identify any compromise of balloon channel during fenestration. Care was taken to apply the punch biopsy 180 degrees from the balloon fill channel, 1.0 cm proximal to the catheter balloon. The catheter was made wet to minimize friction between punch biopsy and latex catheter. The punch biopsy was twisted and gently advanced through the lateral wall of the catheter until the catheter lumen was reached, creating a fenestration.

A sequence of 2.0 mm, 3.0 mm, and 4.0 mm Miltex^®^ punch biopsies (2.0 mm: 33-38; 3.0 mm: 33-38; 4.0 mm: 33-38 respectively), were utilized to place a single fenestration in a Bard 18 Fr 5 cc balloon Foley catheter 1.0 cm proximal to the balloon (Figures 1-2). One fenestration was performed in two sets of catheters in order to provide two catheters for each punch size to increase the validity of the results.

**Figure:**
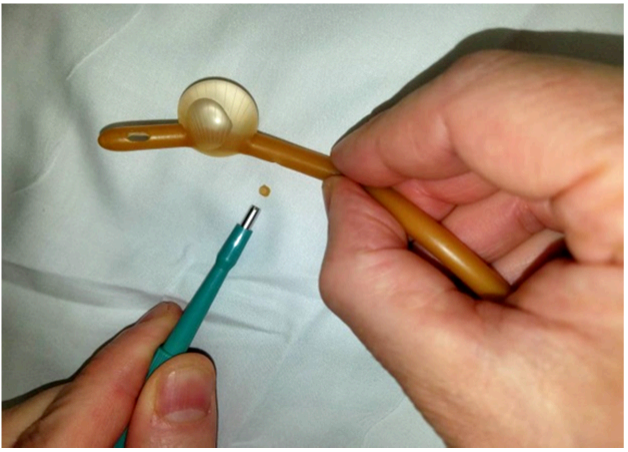
Figure 1 Making the Auxiliary Drainage Hole

**Figure:**
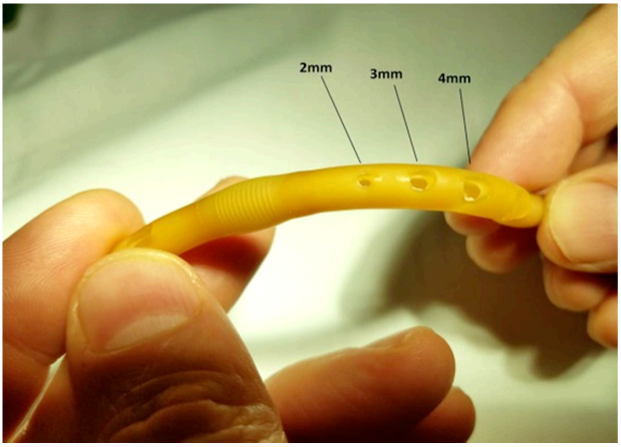
Figure 2 Comparing Sizes of Auxiliary Drainage Holes

A plastic bowl with a hole drilled in the bottom with Foley catheter sealed in the hole was used. (Figure 3) Waterproof tape was applied around the Foley at the base of the bowl. An S-curve was made in the Foley to ensure that any leakage around the Foley would not collect in the graduated cylinder. The authors did not observe any leaking. The new fenestration was held at a predetermined height of 18 cm to standardize the experiment for all catheters. The Foley was clamped distal to the fenestration at all times to ensure that the fluid collected was exclusively through the fenestration. Drainage commenced with filling of the bowl with water and the timer was stopped when the graduated collection cylinder reached 100 ml.

**Figure:**
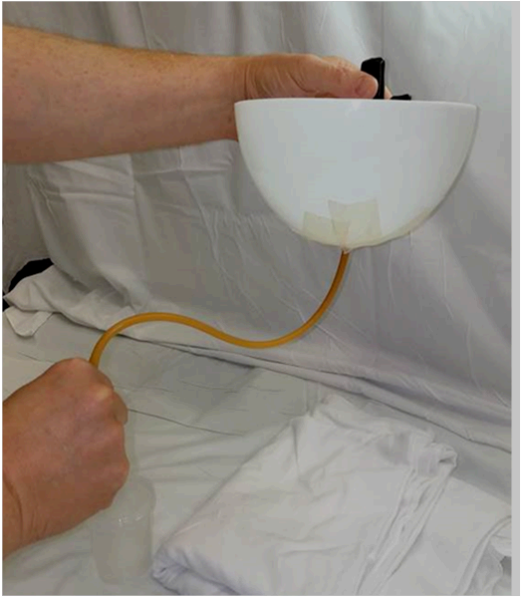
Figure 3 Drainage Measurement System

In addition to determining the rate of drainage for each respective fenestration size, the structural integrity of each Foley catheter was evaluated visually and a binary system was established classifying the 16 Fr and 18 Fr Foley catheter as having “compromised structural integrity” or “not compromised structural integrity.” (Figures 4-5) This was achieved by visually evaluating the effects of the biopsy size on the structural integrity of the system. When bending the catheter, if buckling of the catheter occurred, this was deemed to constitute compromised structural integrity of the catheter.

**Figure:**
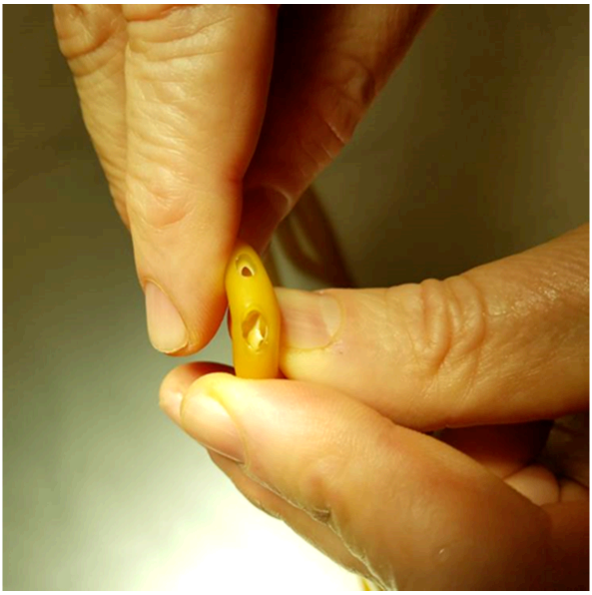
Figure 4 Structural Integrity Test

**Figure:**
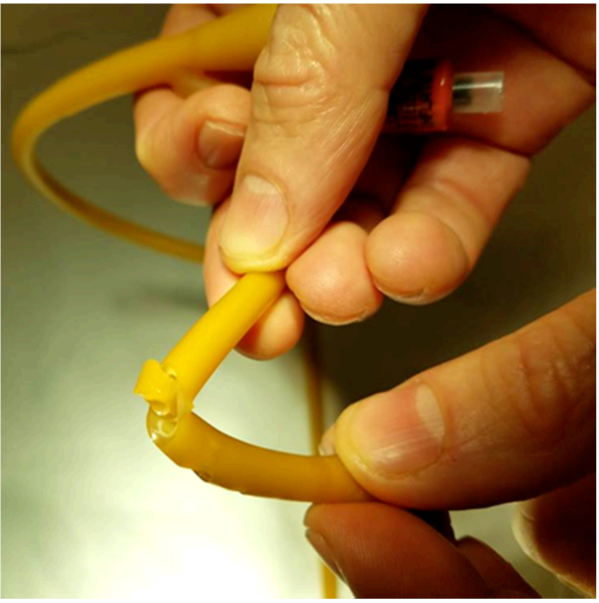
Figure 5 Failing Structural Integrity Test

## RESULTS

As shown in Table 1, the 3.0 mm punch biopsy hold provided superior drainage compared to a 2.0 mm punch biopsy in the 18 Fr Foley catheter. While the 4.0 mm punch biopsy provided greater rate of drainage than the 3.0 mm punch biopsy, there was greater structural compromise evident by buckling of the Foley catheter. This resulted in failure of the binary system of compromised versus not compromised structural integrity. In fact, the experimental arm using 16 Fr Foley catheter was abandoned due to an unacceptable high failure rate. The 2.0 mm and 3.0 mm punch biopsy fenestration resulted in no structural compromise of the Foley catheter.

**Table:** Table 1 Mean Fenestrated Catheter Drainage Rate

	**2mm**	**3mm**	**4mm**
**ml/hr.**	2,094	3,605	9,330
**SD +/-**	747	307	484

The 3.0 mm fenestration provided more than two and a half times the rate of drainage compared to the 2.0 mm. During periods of high patient urine output, this drainage rate would be clinically significant and adequate to minimize the pressure that would increase their risk for AL.

A wide standard deviation occurred in the drainage rate of the 2.0 mm punch biopsy hole, likely due to variability in size of holes created using such a small punch biopsy and our visual observation that the 2.0 mm biopsy was small enough so that the inner lining of the catheter did not cut as smoothly as the 3.0 mm biopsy.

## DISCUSSION

Prostate cancer is the most common oncologic surgery for urologists^8^ and the majority of patients who undergo surgery for prostate cancer experience treatment-related side effects. These side effects can be short term or long term, and that can significantly impact their quality of life.^9^ The portion of the surgery suturing the bladder neck to the urethra to form the anastomosis is considered the most technically challenging with urine AL at this site occurring at a rate of 0.3-15.4% of all RP cases.^2^

Fenestrated catheters have been used for decades in management of AL urologic conditions with multiple publications to support their selective usage. To our knowledge, however, the optimal size of catheter fenestrations has not been systematically evaluated. This low pressure drainage system was first described in 1973 when Turner-Warwick used a fenestrated catheter with multiple holes to drain urethral exudate and hematoma after urethral stricture repairs ^10^ and then later after pelvic fracture.^11^

Reports as early as 2005 showed post robotic assisted laparoscopic prostatectomy (RALP) anastomotic leaks managed by replacing a standard catheter with fenestrated catheter increased the urine drainage per Foley between 1500-2120 ml/day immediately following catheter changes.^5,6^ This finding indicates that urine was flowing out of the catheter rather than into the extra vesical space through the leak.

The only prospective study, a 2014 randomized control trial of standard catheter vs. fenestrated catheters, showed a fenestration made 1 cm proximal to balloon had significantly less AL rates at postop Day 7. In addition, the fenestrated group had less catheter-related side effects (8/125 (6.4%) vs. 3/125 (2.4%)). Patients without leakage tended towards faster recovery of continence (68% at three months) over patients with AL (59% at three months), but this was not statistically significant (p = 0.49), perhaps due to sample size.^7^

Risk factors for AL include history of pelvic radiation, abnormal anatomy, large prostate, post-surgical hematoma formation, obesity, previous prostatic surgery, difficult anastomosis, an anastomosis under tension, blood loss and postoperative urinary tract infection. Although some of these risk factors will be known preoperatively, it is often not until the surgical procedure when the anastomosis work has begun that surgeons will discover there is a less than optimal bladder-urethral anastomosis. In these situations, surgeons have limited options.

All post RP patients have an indwelling Foley catheter which allows the anastomosis to heal with minimal bladder distention reducing strain on the new anastomosis. Unfortunately, indwelling catheters are colonized by bacteria at a rate of about 5% per day; meaning by day 10, 50% of patients with an indwelling urethral catheter will have colonization in the urinary tract.^11^ Post RP patients normally have a catheter left in place for seven to ten days. In numerous reports, the length of time for maintaining this catheter has been found to progressively decreased from 21-30 days^12^ to 14-21 days^13,14^ and more recently to between four and seven days.^8,15,16^

When an AL occurs, this urinary colonization can be catastrophic as this bacteria filled urine leaks out of the bladder. Urine is irritating to intra-abdominal organs and the peritoneal lining and if bacteria are present, the risk for infection is a significant concern that can cause sepsis, abscess formation and further tissue and organ damage.^4^

One significant urologic point to emphasize is that urine leaking into the peritoneal cavity is materially different than in the extra peritoneal space. Urine is caustic and can cause peritonitis when in contact with intraperitoneal organs whereas urine in the extra peritoneal space is not generally as clinically significant. While peritonitis does not occur, complications such as infected urinomas (i.e., inflammatory response in peri-renal fat) can happen if not adequately drained. For example, a bladder perforation that leaks urine intraperitoneal must be repaired emergently whereas an extra peritoneal leak is non-operative.

One key change in urologic practice during recent years is that the majority of RP surgeries are now RALP procedures which already violate the peritoneal cavity and may allow a urine leak to enter the cavity. An open prostatectomy does not normally violate the peritoneal cavity and any leak remains extra peritoneal. A urine leak is not as clinically significant in these patients because urine does not contact the peritoneum or intra-abdominal organs and therefore there is no risk of peritonitis or ileus. AL complications are much more common in open prostatectomy, with one series from 2011-2013 showing a >50% leak rate at postoperative Day 7 on cystogram.^17^

Several key findings of our experiment were evident. First, it is important to use the proper size Foley catheter (18 Fr) and proper size punch biopsy (3.0 mm). Furthermore, it is essential to apply the punch biopsy to a pre-moistened Foley to minimize drag of the latex by the twisting motion of the punch biopsy. When the Foley was dry in our experiment, the punch biopsy did not easily incise the rubber surface of the catheter–the rubber twisted with the twist of the punch biopsy. Additionally, we also found it important not to compress the Foley while twisting the punch biopsy, as that increased the chances of punching through the far side inner wall and causing balloon rupture.

## CONCLUSIONS

A 3.0 mm punch biopsy can be used to fenestrate a moistened 18 Fr Foley on the side opposite the balloon channel 1.0 cm. proximal to the Foley balloon to increase Foley drainage. The 3.0 mm punch did not compromise the structural integrity of the 18 Fr Foley catheter. Use of punch biopsy devices larger than 3.0 mm may compromises the structural integrity of 18 Fr catheters. The 2.0 mm punch biopsy provides lesser drainage rates and is thus considered suboptimal. This can be applied when AL occurs after radical prostatectomy and we recommend considering this method in the setting of RP with patients with a history of pelvic radiation, abnormal anatomy or a tenuous anastomosis.

### Conflict of Interest

The authors declare no conflict of interest.

## References

[ref-3411] American Cancer Society (2017). Prostate cancer.

[ref-3412] Halpern J.A., Shoag J.E., Artis A.S., Ballman K.V., Sedrakyan A., Hershman D.L.. (2017). National trends in prostate biopsy and radical prostatectomy volumes following the US Preventive Services Task Force guidelines against prostate-specific antigen screening. JAMA Surgery.

[ref-3413] Williams T.R., Longoria O.J., Asselmeier S., Menon M. (2008). Incidence and imaging appearance of urethrovesical anastomotic urinary leaks following da Vinci robotic prostatectomy. Abdom Imaging.

[ref-3414] Tyritzis S.I., Katafigiotis I., Constantinides C.A. (2012). All you need to know about urethrovesical anastomotic urinary leakage following radical prostatectomy. J Urol.

[ref-3415] Diamand R., Obeid W.A., Accarain A., Limani K., Hawaux E., Velthoven R.. (2017). Management of Anastomosis Leakage Post-RALP: A Simple Trick for a Complex Situation. Urol Case Rep.

[ref-3416] Kylmälä T., Kaipia A., Matikainen M. (2005). Management of prolonged urinary leakage at the urethro-vesical anastomosis. Urologia Internationalis.

[ref-3417] Riikonen J., Kaipia A., Matikainen M., Koskimäki J., Kylmälä T., Tammela T.L. (2014). Side-fenestrated catheter decreases leakage at the urethrovesical anastomosis after robot-assisted laparoscopic radical prostatectomy. Scand J Urol.

[ref-3426] Patel R., Lepor H. (2003). Removal of urinary catheter on postoperative day 3 or 4 after radical retropubic prostatectomy. Urol.

[ref-3418] Haglind E., Carlsson S., Stranne J., Wallerstedt A., Wilderäng U., Thorsteinsdottir T.. (2015). Urinary incontinence and erectile dysfunction after robotic versus open radical prostatectomy: a prospective, controlled, nonrandomised trial. Europ Urol.

[ref-3419] Turner‐Warwick R. (1973). Observations on the treatment of traumatic urethral injuries and the value of the fenestrated urethral catheter. Brit J Surg.

[ref-3420] Turner-Warwick R. (1977). A personal view of the immediate management of pelvic fracture urethral injuries. Urol Clin North Amer.

[ref-3422] Walsh P.C., Donker P.J. (1982). Impotence following radical prostatectomy: insight into etiology and prevention. J Urol.

[ref-3423] Walsh P.C. (2002). Walsh technique. Abstracts of First Radical Prostatectomy World Summit 2002.

[ref-3424] Souto C.A., Telöken C., Souto J.C., Rhoden E.L., Ting H.Y. (2000). Experience with early catheter removal after radical retropubic prostatectomy. J Urol.

[ref-3425] Souto C.A., Rhoden E.L., De Conti R., Chammas Jr. M., Laste S.E., Fornari A., Ribeiro E.P., Scholl L., Teloken C., Souto J.C. (2004). Urethral catheter removal 7 or 14 days after radical retropubic prostatectomy: clinical implications and complications in a randomized study. Revista do Hospital das Clínicas.

[ref-3427] Santis W.F., Hoffman M.A., Dewolf W.C. (2000). Early catheter removal in 100 consecutive patients undergoing radical retropubic prostatectomy. BJU Intnl.

[ref-3428] Cormio L., Di Fino G., Scavone C., Maroscia D., Mancini V., Ruocco N., Bellanti F., Selvaggio O., Sanguedolce F., Lucarelli G., Carrieri G. (2016). Prognostic factors for anastomotic urinary leakage following retropubic radical prostatectomy and correlation with voiding outcomes. Medic.

